# The effect of wheat germ on the chemical properties and fatty acids of white cheese during the storage time

**DOI:** 10.1002/fsn3.1370

**Published:** 2019-12-27

**Authors:** Asya Çetinkaya, Fatih Öz

**Affiliations:** ^1^ Department of Food Engineering Faculty of Engineering Architecture Kafkas University Kars Turkey; ^2^ Department of Food Engineering Faculty of Agriculture Atatürk University Erzurum Turkey

**Keywords:** chemical properties, fatty acids, wheat germ, white cheese

## Abstract

In the present study, it was aimed to support the white cheese structure produced from cow's milk by adding wheat germ and to determine the changes in fatty acids and chemical composition of this additive storage time. The samples were taken on the 1st, 15th, and 30th days of storage, and some chemical properties and fatty acids were analyzed. The use of wheat germ in the production of white cheese had a significant effect (*p* < .05) on the fat, protein, salt, total acidity (% l.a) and dry matter properties of the cheese. It was determined that the most common fatty acids in the cheese samples were palmitic, oleic, stearic, and myristic acids. Wheat germ is a significant source due to its high protein content. Therefore, it can be expressed as an auxiliary raw material for the development of nutritional and functional properties in cheese production.

## Graphical abstract

We investigated the differences in the chemical properties and fatty acid components of wheat germ addition during the ripening period of white cheese. According to the results of the study, it is recommended to use wheat germ as an alternative to enriching the nutritional content of cheese which is an important dairy product.

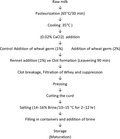

## 1 INTRODUCTION

White cheese is a soft or semihard cheese class of raw cows, sheep's milk, or a mixture of these milk obtained by using the traditional Turkish cheese ripened in brine. White cheese production in Turkey is done in home, family type businesses, local dairies, and factories. In the traditionally produced cheeses, starter culture is generally not used (Öner, Karahan, & Aloğlu, 2006).

Although the characteristics of white cheese depend on the titration acidity and the amount of salt, the final products of lipolysis and proteolysis are effective in the formation of these properties (Güler & Uraz, 2004).

As well as its characteristic of being easily digested, cheese is also rich in vitamins, minerals, and high‐quality protein (Öksüztepe, Karatepe, Özçelik, & İncili, 2013). The rate of digestion increases as a result of hydrolysis of proteins during ripening of cheeses and thus helps digestion of other foods (Demirci, 1990).

The chemical composition of wheat germ was determined as 28.5% protein, 14.0% starch, 11.7% moisture, 10.4% fat, 7.5% cellulose, 6.8% hemicellulose, and 4.5% ash (Avcıoğlu, 2014).

Wheat germ content contains high levels of lecithin, essential fatty acids, unsaturated fatty acids (oleic, linoleic, and alpha‐linolenic), proteins and minerals zinc, manganese, and chromium among minerals (https://www.rafinera.com/blog/diyetisyen-kosesi/bugday-ruseymi-vefaydalar, 2019; Özcan et al., 2013).

The biological value of the proteins found in wheat germ (%23) is similar to animal (meat %16–22, milk %3.5, and cheese %16–30 proteins) (https://www.fitekran.com/besin-degeri/ruseym/ 2019; Arslan, 2002; İnal, 1990).

Wheat germ, produced with or without oil, is offered for sale in plastic bags or jars at the places in Europe and the USA where healthcare and dietary products are sold. These products can be added to soups, milk, and yoghurt and consumed with breakfast cereals by using various sweeteners. Wheat germ is used as filler and enrichment agent for various foodstuffs, especially bread (Çakmaklı, Köse, & Kemahlıoğlu, 1995; Kahveci & Özkaya, 1990; Türker, Elgün, & Keskinoğlu, 1996).

In India, bread and biscuits were made by adding commercially obtained wheat germ (stabilized directly and by being roasted, steamed, degreased). It was determined that the protein content of breads increased by 3% when 15% of wheat germ was added in bread making (Shurpalekar, Rao, Kumar, & Rao, 1979).

Sümbül and Tanju (1982) They stated that bread, biscuits, pasta, cakes, foods for breakfast, and cereals can be made with wheat germ, and it can also be used in houses to sprinkle on soup and salad or presented for consumption to be used directly for omelette, meatballs, and various forms.

It was stated that proteolytic preparations obtained through an extraction from wheat germ can be used in the ripening of meats and that these preparations improve the taste as well as softening the meat (Flaczyk & Kaminski, 1978).

In a study, in which cookies were made by using three different wheat germ, flour combination, two different antioxidant types (ascorbic acid, BHT), and five different oil ratios, it was stated that hte cookies with the ratios of 70 wheat germ: 30 flour and 60 wheat germ: 40 flour gave the desired results the best according to the technologic and sensory results (Avcıoğlu, 2014).

Mohamed, Seleet, Abd El Khalek, and Fathy (2015). They stated that the Labneh cheese produced by adding wheat germ extract had a higher dry matter level and higher hardness than the control group and also had an effect on the taste and flavor of the product.

Basiony (2013) stated that as a result of cheese production, which was made by adding such ingredients as wheat seeds, oat, saccharin fiber, and barley, an increase in cheese yield and in the amounts of protein, ash, and fatty acids was achieved as well as a decrease in clotting time.

Cheese production by adding various components is common in many countries. For example, in Greece, Italy, Spain, and Portugal, cheeses made with hazelnut, fruit, vegetable, and spice crops, soybean products added to Japan, China, Korea, and other countries, cheeses made with isolates, and vegetable ingredients in Southeast Asia and Western European countries and in America like cheeses (Zakharova, 2014).

Nahla and Makarim (2018) Studies; wheat seeds in soft cheeses also improve the sensory properties developed.

The aim of this study was to produce white cheese by adding different ratios of wheat germ to cow's milk and to evaluate the effect of this additive on the chemical composition and fatty acid components of this product and the changes in these components storage time.

## 2 MATERIALS AND METHODS

### 2.1 Material

**Figure 1:**
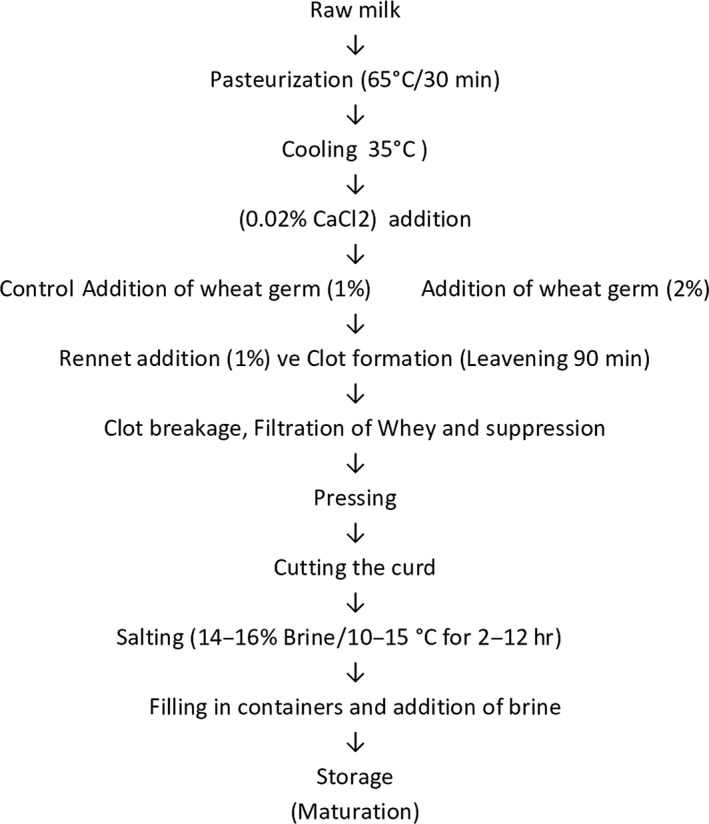
White cheese production flowchart

White cheese production was carried out at the Research Laboratory of Kafkas University, Food Engineering Department. In the production, raw cow milk was obtained from the milk factories located in the industrial zone of Kars province. Natural Renna liquid commercial rennet (rennet) at 1/16.000 strength was used as cheese enzyme. After the rennet was diluted with pure water at 1/10 ratio, it was added to milk to be used in the production of cheese. Wheat germ was purchased as milled inside package from the markets. 5 kg glass jars were used as the packaging material. Production was done in two replications. For the production, milk was divided into three parts as control group without any added wheat germ and the groups with 1% and 2% wheat germs. On the 1st, 15th, and 30th days of storage, the analyses of white cheese samples were made. The white cheese production was carried out in accordance with the flow chart given in Figure 1.

### 2.2 Methods

Raw wheat germ was added in 1% and 2% concentrations during the production of white cheese. Wheat germ, cow milk, and processed cheeses’ chemical content and fatty acid profile were investigated.

#### 2.2.1 Chemical analyses

Ash, total nitrogen, and fat content of wheat germ samples were determined according to AOAC (2007).

Lipid assay in cheese samples was determined according to Gerber method (Turkish Standards Institution, 1978). Amount of dry matter was determined with the gravimetric method. Titratable acidity was determined with the alkali titration method, and results were expressed as lactic acid % (Turkish Standards Institution, 2006). The amount of protein was determined using the micro‐Kjeldahl method, and it was calculated by multiplying the total nitrogen amount found by the Kjeldahl method by a factor of 6.38 and expressed as % (International Dairy Federation, 1993). Salt ratios were determined according to the Mohr titration method, and the results were expressed as % (Turkish Standards Institution, 1989, 2006).

#### 2.2.2 Determination of fatty acid composition

Fatty acid composition of the examples was determined according to the fatty acid methyl ester method (FAME) (AOAC 996.01) (Satchithanandam, Fritsche, & Rader, 2001). According to the method, approximately 0.1 g of fat, which was obtained as a result of the lipid assay, was rinsed with 10 ml of n‐hexane and re‐mixed with 0.5 ml of 2 N methanolic potassium hydroxide solution. After having been kept in a dark environment from for 1–2 hr, 1 µl was taken from the supernate and directly injected into the gas chromatograph.

##### GC Conditions for fatty acid composition analysis

Fatty acid methyl ester composition in fat was analyzed by using Restek RTX‐2330 capillary column (60 m, 0.25 mm i.d.0.1 µm film thickness; Bellefonte, PA, USA) and flame ionization detector (FID) in a Shimadzu brand gas chromatograph (model QP2010 Plus). The device was given 1 µl of injection volume from the sample. Column furnace temperature was increased to 240°C with 4°C/min increase after being kept for 3 min at 100°C and was programed to wait 18 min at the final temperature. The injection temperature was set to 250°C and the detector temperature to 255°C. Helium was used as carrier gas in the device. Injection split ratio was used at 1:80 ratio. To control the GC/FID system, LabSolution computer program was used and FAME mix 37 standard (Restek) as standard. FAME peaks were specified by comparing the chain lengths and retention times of the fatty acids specified in FAME standard.

#### 2.2.3 Statistical analysis

In the evaluation of the obtained results and standard deviation of the samples were determined using SPSS 18.0.0 package program. 

## 3 RESULTS AND DISCUSSION

Table 1 shows the values of whole‐fat cow milk and wheat germ used in experimental white cheese production. The fat, protein, ash, total dry matter, and total acidity (% l.a) values of cow milk were 3.7, 3.2, 0.63, 12.80, and 0.16, respectively. The ratios determined in milk are similar to those determined by Nahla and Makarim (2018) in milk used in soft cheese production.

**Table 1:** Chemical composition of whole raw bovine milk and wheat germ

Compound	Raw milk	Wheat germ
Fat (%)	3.7	9.64
Protein (%)	3.2	33
Dry matter (%)	12.8	–
Total acidity (%l.a)	0.16	–
Ash (%)	0.63	4

The protein and ash values of wheat germ are similar to those found by Abbas, Hussein, Seleet, Bayoumi, and Abd El‐Aziz (2015) but lower than those determined by Nahla and Makarim (2018). The differences in the chemical components of wheat germ might have been due to the type of the wheat and the differences in the systems and methods in the mills during processing.

Having high protein quality and being rich in vitamins make it important to add wheat germ in foodstuffs as a food additive. It has been stated by many researchers that wheat germ is a very suitable source for the enrichment of foodstuffs with proteins and vitamins due to its high nutritional value as well as its good taste and flavor (Çakmaklı et al., 1995; Kahveci & Özkaya, 1990; Türker et al., 1996).

Table 2 shows the chemical composition (A, B, and C) of white cheese samples produced by adding the control group and wheat germ in different ratios. Dry matter ratios of cheese samples were higher than those determined by Mohamed et al. (2015) in Labneh cheese, and were found to be lower than Erdem and Patır (2017) Tulum cheese, Yılmaz, Gürsoy, Gökçe, and Ertan (2015) in Labne cheese and Özdemir, Yangılar, and Özdemir (2013) the value determined in Karın Kaymağı cheese. It was found that in the study of Weiwei et al. (2016), the fat ratios of cheese samples with wheat germ were lower than the soft cheeses made by Nahla and Makarim (2018) by adding wheat germ to the control group and containing 1% wheat germ group, and higher than containing 2% wheat germ cheese.

**Table 2:** Mean values of some chemical and physical properties obtained from the experimental cheeses storage time (%)

	*n*	Fat	Total acidity (% l.a)	Protein	Dry matter	Salt
Experimental cheeses						
A	6	10.75 ± 0.42b	0.40 ± 0.10b	15.26 ± 0.36b	36.69 ± 0.94c	2.48 ± 0.80
B	6	13.58 ± 0.88a	0.77 ± 0.21b	16.47 ± 0.39b	39.09 ± 0.54b	3.73 ± 0.48
C	6	13.93 ± 0.50a	1.00 ± 0.09ab	18.09 ± 0.63a	42.03 ± 0.61a	3.85 ± 0.51
Sign		**	*	**	**	ns
Storage time (day)						
1	6	11.60 ± 0.53b	0.38 ± 0.12b	15.33 ± 0.39b	37.24 ± 1.21b	1.74 ± 0.55a
15	6	12.83 ± 0.72ab	0.68 ± 0.13b	16.72 ± 0.54ab	39.61 ± 0.80ab	3.45 ± 0.07b
30	6	14.26 ± 0.83a	1.11 ± 0.12a	17.77 ± 0.66a	40.96 ± 0.93a	4.87 ± 0.24c
Sign		*	**	*	*	**

Note

A: control, B: %1 wheat germ, C: % 2 wheat germ. Sign, significance and ns, not significant (*p* > .05). Different letters (a,b,c) indicate significant differences (*p* < .05), (*p* < .01)

* *p* < .05

** *p* < .01

Protein values of cheese samples are lower than the values determined by Weiwei et al. (2016) and higher than those determined by Nahla and Makarim (2018) in soft cheese. The high protein content is due to the increase in solids concentration by adding wheat germ to milk. The addition of wheat germ in different ratios in white cheese production has led to an increase in protein, fat and dry matter content in cheese samples.

Fat, dry matter, total acidity (% 1.a), protein, and salt ratios increased during 30 days of storage time in white cheese samples. As a result of the statistical evaluation, this increase was found to be statistically significant ((*p* < .05, *p* < .01) (Table 2).

It has been reported that fatty acids are important components affecting the taste of white cheese. It is thought that free fatty acids are produced during lactation by lactic acid bacteria and secondary flora in this type of cheese. For balanced flavor development, it is important that fat hydrolysis coexist with the hydrolysis of other milk components (Georgala, Kandarakis, Kaminarides, & Anifantakis, 1999). The increase in the amount of fatty acids with increasing storage time can be explained by the effect of bacterial lipases. With the breakdown of bacterial cells in cheese over time, enzymes are released and can contribute to lipolysis. Lipases in cheese are intracellular lipases that are released by lactic acid bacteria through bacterial lipolysis and pregastric lipase. Lactic acid bacteria are responsible for the formation of significant free fatty acids in cheese (Collins, McSweeney, & Wilkinson, 2003). Fatty acids directly affect the formation of flavor in cheese.

Table 3 shows the amount of fatty acids in the cheese samples. Fatty acid values of cheese samples increased during 30 days of storage period. This increase was found to be significant in ΣSFA (saturated) and ΣPUFA (polyunsaturated fatty acids) fatty acids (*p* < .05) and insignificant in ΣMUFA (monounsaturated) fatty acids (*p* > .05).

**Table 3:** Fatty acid composition of experimental cheeses during the storage time (%)

	n	Palmitic acid	Stearic acid	Myristic acid	Other saturated fatty acids	ΣSFA	Oleic acid	Other monounsaturated fatty acids	ΣMUFA	Linoleic acid	Other polyunsaturated fatty acids	ΣPUFA
Experimental cheeses												
A	6	32.56 ± 0.30b	13.30 ± 0.76a	9.68 ± 0.15b	9.75 ± 0.37b	65.81 ± 0.75b	27.92 ± 0.98a	3.28 ± 0.58	31.38 ± 0.75a	1.94 ± 0.24	0.65 ± 0.20a	2.80 ± 0.59b
B	6	33.08 ± 0.51a	12.59 ± 0.43ab	10.15 ± 0.19b	11.34 ± 0.02a	66.66 ± 0.71ab	27.00 ± 0.75ab	3.71 ± 0.02	30.44 ± 0.96a	2.12 ± 0.23	0.14 ± 0.01c	2.90 ± 0.49ab
C	6	32.71 ± 1.16b	12.29 ± 0.27b	10.68 ± 1.36a	11.32 ± 0.27a	67.02 ± 2.69a	26.06 ± 1.27b	4.02 ± 0.13	29.78 ± 1.39b	1.91 ± 0.53	0.40 ± 0.16b	3.15 ± 2.09a
Sign		*	*	**	**	*	*	Ns	*	ns	*	*
Storage time (Day)												
1	6	32.86 ± 0.50	12.50 ± 0.49b	10.26 ± 0.44ab	11.01 ± 0.47a	66.63 ± 0.97ab	23.75 ± 0.86	7.05 ± 0.08	30.80 ± 0.93	1.82 ± 0.19	0.55 ± 0.24b	2.37 ± 0.64b
15	6	32.93 ± 0.70	13.05 ± 0.98a	10.56 ± 1.29a	10.46 ± 0.91b	67.00 ± 2.07a	26.62 ± 1.75	3.56 ± 0.06	30.18 ± 1.73	2.04 ± 0.43	0.78 ± 0.20b	2.82 ± 0.64ab
30	6	32.56 ± 1.01	12.62 ± 0.26b	9.80 ± 0.84b	10.68 ± 0.45b	65.67 ± 1.66b	27.02 ± 1.04	3.59 ± 0.10	30.61 ± 1.15	2.11 ± 0.38	1.55 ± 0.93a	3.66 ± 1.78a
Sign		ns	**	*	*	*	Ns	ns	ns	ns	*	*

Note

Sign, significance and ns, not significant (*p* > .05). Different letters (a,b,c) in the same line refers significant differences between the averages *(*p* < .05).

* *p* < .05.

** *p* < .01.

Butyric, caproic, and capric acid amounts of saturated fatty acids detected in cheese samples were lower than those determined by Kinik, Gursoy, and Seckin (2005) in white cheese samples and higher than the lauric, myristic, palmitic, and pentadecanoic acid values, close to the stearic acid value and similar to the lauric acid value that Abo‐Elwafa, Foda, and Aly (2015) determined in white soft cheese samples and between the myristic, palmitic, and stearic acid values in range. In addition, the stearic and palmitic acid values were lower than those that Nahla and Makarim (2018) determined in soft cheese samples added with wheat germ.

It is stated that wheat germ oil contains high levels of unsaturated fatty acids. (Wang & Johnson, 2001). The amount of oleic and linoleic acids detected in cheese samples made by adding different amounts of wheat germ was lower than the values determined by Nahla and Makarim (2018) and higher than the values that Abo‐Elwafa et al. (2015) determined in white soft cheese and Kinik et al. (2005) in white cheese. The amount of palmitoleic acid of cheese samples was higher than the value that Kinik et al. (2005) determined in white cheese, while the myristoleic acid content is similar and the cis‐11‐eicosenoic acid content is high. Due to the high content of essential fatty acids, especially unsaturated fatty acids, (oleic, linoleic, and alpha‐linolenic) in wheat germ, the amount of fatty acids increased in relation to the addition of wheat germ to cheese in comparison to the control group.

Generally, milk fat contains about 66% saturated (SFA) (57.4% palmitic, 21.6% myristic, and 17.6% stearic), 30% monounsaturated (MUFA), and 4% polyunsaturated fatty acids (PUFA) (López‐Expósito, Amigo, & Recio, 2012). About 66.84% of the total fatty acids determined in the cheese samples made by adding wheat germ were saturated fatty acids (49.21% palmitic, 15.58% myristic, and 18.61% stearic), and 33.13% of them were monounsaturated (MUFA) and 3.02% consisted of polyunsaturated fatty acids (PUFA).

## 4 CONCLUSIONS

According to the data obtained as the result of this study, the addition of wheat germ to cheese has increased the ratio of protein, fat, dry matter, and fatty acids. This study shows that the cheese produced by adding wheat germ to cheese has a higher solids level than the control group. It can be said that wheat germ high protein content can be an auxiliary raw material in cheese production for improving nutritional and functional properties thanks to its balanced amino acid distribution, containing essential fatty acids and being an important fiber source. In addition, further studies may be recommended for the use of wheat germ as an alternative for enriching the nutritional content of cheese, which is an important dairy product.

## CONFLICT OF INTEREST

The authors declare no conflict of interest.

## ETHICAL APPROVAL

This study has nothing to do with human and animal testing.
